# Endothelial Injury Following CAR-T Cell Immunotherapy for Hematological Malignancies

**DOI:** 10.3390/cancers17172876

**Published:** 2025-09-01

**Authors:** Christos Demosthenous, Paschalis Evangelidis, Athanasios Gatsis, Ioannis Mitroulis, Sofia Vakalopoulou, Anna Vardi, Stefania Bountoura, Ioanna Sakellari, Eleni Gavriilaki

**Affiliations:** 1 BMT Unit, Hematology Department, George Papanicolaou General Hospital, 57010 Thessaloniki, Greece; christosde@msn.com (C.D.); anna.vardi@yahoo.com (A.V.); stef.bountoura@gmail.com (S.B.); ioannamarilena@gmail.com (I.S.); 2 Second Propedeutic Department of Internal Medicine, Hippocration Hospital, Aristotle University of Thessaloniki, 54642 Thessaloniki, Greece; pascevan@auth.gr (P.E.); athanasiosgatsis@gmail.com (A.G.); svakalop@auth.gr (S.V.); 3 Translational Research and Laboratory Medicine Unit, First Department of Internal Medicine, University Hospital of Alexandroupolis, Democritus University of Thrace, 68100 Alexandroupolis, Greece; imitroul@med.duth.gr; 4 Department of Hematology, University Hospital of Alexandroupolis, Democritus University of Thrace, 68100 Alexandroupolis, Greece

**Keywords:** CAR-T, complement, CRS, endothelium, ICANS, lymphoma, myeloma

## Abstract

Chimeric antigen receptor-T (CAR-T) cell immunotherapy constitutes a cornerstone in the management of patients with relapsed/refractory B-cell lineage lymphoid malignancies. Toxicities such as cytokine release syndrome (CRS), immune effector cell-associated neurotoxicity syndrome (ICANS), and hematotoxicity (ICAHT) have been recognized in the post-infusion period. The initial interplay between CAR-T cells and tumor cells, followed by cytokine release and the bystander activation of the innate immunity cells, result in endothelial cell injury. In the current review, the ongoing research regarding endothelial injury in CAR-T cell recipients is summarized. Various markers of endothelial injury have been investigated in CAR-T cell recipients, including markers of complement activation, such as soluble C5b-9, endothelial dysfunction (angiopoietin-2, VCAM1, ICAM-1), inflammation, and thrombosis (von Willebrand antigen, ADAMTS13, thrombomodulin). The expression level of these endothelial injury markers has been identified as impaired in CAR-T cell recipients, not only when compared with healthy controls but also among patients with severe CRS/ICANS and those with mild toxicities or without toxicities. Furthermore, the Endothelial Activation and Stress Index (EASIX) and modified versions of this score, calculated in the pre- and early post-infusion period, seem to predict development of severe toxicities, ICAHT, and, thus, poor overall survival in CAR-T cell patients. More data concerning the role of these endothelial injury markers and clinical outcomes in CAR-T cell settings are essential.

## Simple Summary

Chimeric antigen receptor-T (CAR-T) therapy has become, nowadays, a standard of care for patients with relapsed/refractory lymphomas, myeloma, and acute lymphoblastic leukemia. Cytokine release syndrome (CRS) and neurotoxicity, associated with immune dysregulation and cytokine storm, result in endothelial injury and increased blood–brain barrier permeability. Markers indicative of complement pathway activation, inflammation, and thrombosis have been found to increase in the post-infusion period. Additionally, these markers, in several studies, have been reported to be higher in CAR-T cell recipients who present severe forms of toxicities compared to those with mild or without CRS/ICANS. Endothelial Activation and Stress Index (EASIX) scores have been shown to predict poor outcomes in these patients.

## 1. Introduction

Chimeric antigen receptor-T (CAR-T) cell immunotherapy has revolutionized the treatment landscape for patients with relapsed/refractory (R/R) lymphomas and B-cell acute lymphoblastic leukemia (B-ALL) [1,2]. Currently, four CAR-T cell products—axicabtagene ciloleucel, brexucabtagene autoleucel, lisocabtagene maraleucel, and tisagenlecleucel—are commercially available for R/R B-cell hematological malignancies [3], with two additional products approved by the Food and Drug Administration (FDA) for R/R multiple myeloma (MM) [4]. Beyond hematological malignancies, CAR-T cell immunotherapy is also being explored for the management of patients with autoimmune disorders, such as systemic lupus erythematosus, and solid tumors [5,6].

Real-world evidence confirms the safety and efficacy of CAR-T cell therapy for patients with R/R hematological malignancies [7,8]. Nevertheless, various complications can arise in the post-infusion period [9]. Toxicities such as cytokine release syndrome (CRS) and immune effector cell-associated neurotoxicity syndrome (ICANS) are commonly observed in the early period after CAR-T cell therapy [10,11]. Hematological toxicity, including coagulation dysregulation and cytopenias, also described as immune effector cell-associated hematotoxicity (ICAHT), is an additional adverse event [12]. Other possible complications include tumor lysis syndrome (TLS), infections and sepsis events (bacterial, viral, and fungal), neurocognitive impairment in both the early and late infusion period, and cardiovascular and thromboembolic events [13,14,15,16]. Despite advancements in managing these toxicities, the mechanistic basis implicated in their pathophysiology remains poorly understood. Emerging evidence highlights the critical role of endothelial injury in the pathogenesis of CRS and ICANS pathogenesis, often referred to as “endotheliopathies” [17,18]. Although CAR-T cells have demonstrated a significant role in the management of patients with complex hematological malignancies, CAR-T-related toxicities remain a significant barrier, posing a challenge to their efficacy. Immune system activation is evident in CRS and ICANS, which leads to the release of several proinflammatory molecules from monocytes–macrophages, such as angiopoietin-2 (ANG-2), interleukins, and colony-stimulating factors (GM-CSFs), resulting in endothelial injury, platelet aggregation, increased vascular permeability, and disturbances in the blood–brain barrier (BBB) [10,17]. This review examines the markers of endothelial activation and injury in CAR-T cell recipients following infusion and their associations with toxicity. Given the wide use of CAR-T cellular therapies in everyday clinical practice, a deeper understanding of endothelial injury post-infusion is crucial for identifying relevant biomarkers and developing new prophylactic and therapeutic strategies.

## 2. CRS: Basic Insights

CAR-T cell therapy has shown great promise in treating complex hematological malignancies, but it often comes with challenging toxicities. CRS is the most common systemic toxicity, occurring in 58–94% of patients [19], with some reports indicating incidences as high as 100% [20]. The detailed pathogenesis of CRS remains incompletely understood. Upon recognizing target antigens in vivo, CAR-T cells are rapidly stimulated and secrete large amounts of tumor necrosis factor-a (TNFA-a), interferon-γ (IFN-γ), perforin, and granzyme. Consequently, tumor cell pyroptosis takes place, along with the release of danger-associated molecular patterns (DAMPs). DAMPs can recruit and activate cells of endogenous innate immunity, including dendritic cells and macrophages, amplifying inflammatory responses and increasing the release of proinflammatory cytokines, such as interleukin-6 (IL-6) and interleukin-1β (IL-1β) [20,21]. The cytokines secreted from the activated immune cells (i.e., IL-1, IL-6, TNF-a) induce endothelial cell (EC) activation through mitogen-activated protein kinase/nuclear factor kappa-light-chain enhancer of activated B cell (MAPK/NFκB) signaling pathway, leading to Weibel–Palade degranulation and the release of ANG-2 [10,22], in addition to VWF. ANG-2 displaces angiopoietin-1 (ANG-1) from angiopoietin-1 receptor (TIE-2), resulting in EC-to-cell junction impairment, increased expression of adhesion molecules such as intercellular adhesion molecule-1 (ICAM-1) and vascular cell adhesion protein-1 (VCAM-1), and procoagulant molecule secretion [23].

The disruption of molecular balance and normal EC function is directly correlated with the clinical manifestations of the syndrome. Initially, patients may experience fever within a few hours to a week following CAR-T cell infusion, which might be succeeded by hypotension, sinus tachycardia, impaired cardiac function, increased vascular permeability and capillary leak syndrome manifestations [24]. In severe cases, CRS can escalate to multi-organ dysfunction, associated with reversible toxicities in most cases, but necessitating intensive care interventions. Related manifestations might include constitutional symptoms (e.g., malaise, fatigue), renal deterioration, hepatic and gastrointestinal discrepancies, musculoskeletal toxicity, and hematologic abnormalities [25]. CAR-T cell therapy is rarely complicated by hemophagocytic lymphohistiocytosis (HLH), recently defined as IEC-associated HLH-like syndrome (IEC-HS). IEC-HS is a hyperinflammatory process that is characterized by concurrent CAR-T cell/macrophage activation, cytokine abnormalities, and new-onset or progressive cytopenias, hyperferritinemia, or hypofibrinogenemia with coagulopathy and/or transaminitis [26,27]. Although the IEC-HS mechanism in the setting of CRS is not entirely delineated, severe forms of these entities may further complicate the prognosis [24]. Higher CAR-T cell doses, in vivo CAR-T expansion, and lymphodepleting chemotherapy (LD) containing fludarabine are treatment-related factors predisposing to increased CRS severity, while patient-related factors include a diagnosis of ALL as compared to non-Hodgkin lymphoma (NHL), pre-infusion thrombocytopenia, and baseline elevated markers of endothelial dysfunction [24]. Additionally, features of the infused CAR-T cell product might contribute to the pattern of toxicity. For instance, the costimulatory domain CD-28 may be associated with earlier onset of CRS [24,28].

Multiple grading scales have been used in clinical studies for the assessment of CRS severity and the need for therapeutic interventions. In the American Society for Transplantation and Cellular Therapy (ASTCT) consensus, a grading system has been developed for CRS, as well as for ICANS (Table 1), based on the clinical status of the patient [26]. Various preclinical and clinical studies have provided insights into CRS pathogenesis, thus suggesting potential treatment approaches by targeting crucial molecular determinants of the inflammatory cascade involved in the syndrome development. Symptomatic treatment and tocilizumab, an IL-6 receptor antagonist that has shown remarkable outcomes without interfering with CAR-T cell therapy efficiency, are recommended as first-line treatment options [29]. Corticosteroids are often used for the management of refractory and/or more severe CRS. Siltuximab, anakinra, and etanercept targeting IL-6 molecules, IL-1 receptors, and TNF-a molecules, respectively, have been occasionally utilized as well [24]. In addition, granulocyte–macrophage GM-CSF antibody, lenzilumab, and GM-CSF gene-knockout CAR-T cells are currently being tested along with other agents, such as inhibitors targeting Janus kinase/signal transducers and activators of transcription (JAK/STAT) pathway (e.g., ruxolitinib) and Bruton’s tyrosine kinase (BTK) (e.g., ibrutinib) [30].

## 3. ICANS and Neurotoxicity

Immune effector cell-associated neurotoxicity syndrome (ICANS) is a common complication in the early post-infusion period of CAR-T cell therapy, with an incidence ranging from 15% to 70% [31]. Although ICANS is considered a distinct clinical entity from CRS, these toxicities often coexist and share a common pathogenic background. The interaction between CAR-T cells and host myeloid cells induces cytokine release, leading to the activation of BBB endothelium and increased BBB permeability. This results in the migration of inflammatory cells into the cerebrospinal fluid (CSF) and central nervous system (CNS) [31]. Pinto et al. summarized the effect of several cytokines on BBB function following CD19 CAR-T cell exposure. IFN-γ, secreted by activated CAR-T cells, and GM-CSF, derived from the monocyte/macrophage system, induce the expression of leucocyte adhesion molecule expression (ICAM-1, VCAM-1), promoting monocyte attachment and migration. Activated monocytes and macrophages produce IL-1β, which impairs the BBB by downregulating Sonic Hedgehog (SHH) pathways and, consequently, suppressing the expression of tight junction by astrocytes [32]. Increased ANG-2 release from Weibel–Palade vesicles is a common point of endothelial dysfunction between ICANS and CRS [33].

Neurotoxicity typically occurs in the first 4–10 days after CAR-T cell infusion and often follows CRS development, although it has been sporadically reported to occur independently [29,33]. Symptoms often manifest in a monophasic manner, starting with aphasia, dysgraphia, tremor, and headache, which may progress to cerebral edema, lethargy, agitation, seizures, global aphasia, obtundation, stupor, and coma [34,35]. Seizures, primarily tonic–clonic, often follow the frontal rhythmic intermittent delta activity (FRIDA) pattern on electroencephalogram (EEG) [19]. Imaging findings such as leptomeningeal enhancement, white matter changes, cerebral sulci T2 hyperintensity, and thalamic swelling are non-specific and typically symmetric, corresponding to severe neurotoxicity [34]. However, most ICANS cases do not show MRI abnormalities [34,36]. Factors predisposing to increased ICANS severity include high tumor burden, preexisting neurological symptoms, increased lymphodepleting chemotherapy (LD) intensity, CAR-T cell dose, and in vivo proliferation of infused cells [37,38]. CAR-T cell structural features, such as costimulatory and hinge domains, are also linked to ICANS development [31]. The presence and earlier onset of CRS, along with elevated C-reactive protein (CRP) and ferritin levels, are major risk factors for ICANS, while pretreatment lactate dehydrogenase (LDH) levels correlate with ICANS grading [39,40].

ICANS severity can be assessed using the ASTCT consensus grading scale, which classifies neurotoxicity according to the Immune Effector Cell-Associated Encephalopathy (ICE) score, level of consciousness, the presence and severity of seizures, motor deficits, as well as the presence of signs of increased intracranial pressure (ICP) and cerebral edema (Table 1) [26]. The ICE score is an easily applicable, 10-point neurologic assessment scale evaluating orientation, naming, writing, attention, and the ability of the patient to follow commands [41]. ICANS diagnosis and management involve frequent patient assessment using the ASTCT scale, serial monitoring of laboratory tests (e.g., CRP, ferritin, complete blood count, fibrinogen, prothrombin time, activated partial thromboplastin time), neuroimaging, EEG evaluation, fundoscopy, and aspiration prophylaxis. Treatment is adjusted based on neurotoxicity grade: seizure prophylaxis for grade I, intravenous dexamethasone for grades II and III, and high-dose intravenous dexamethasone for grade IV [42]. Anakinra, silituximab, intrathecal hydrocortisone, cyclophosphamide, and antithymocyte globulin are used in cases of refractory neurotoxicity [30]. Tocilizumab is a monoclonal antibody that cannot cross the BBB [43]. Moreover, the mechanism of action of tocilizumab leads to higher blood levels of IL-6, potentially enhancing CNS exposure to IL-6, which raises concerns for increased CNS toxicity [44]. As it blocks IL-6R in peripheral tissues, it should be used when concurrent CRS exists [43].

## 4. Endothelial Injury Markers in CAR-T Cell Recipients

### 4.1. An Overview of Studies Investigating Endothelial Injury Markers in CAR-T Cell Recipients

Endothelial injury plays a pivotal role in the pathogenesis of CRS and ICANS [45]. CRS and ICANS have been described as “endotheliopathies,” meaning that endothelial injury and activation play a major role in the pathogenesis of these syndromes [46]. It has been supported that the pathophysiology of CRS and ICANS shares the same pathogenic background with endothelial injury syndromes observed post-allogeneic hematopoietic stem cell transplantation (allo-HCT), such as transplant-associated thrombotic macroangiopathy (TA-TMA) [47]. Several studies have examined the association between endothelial damage and the incidence and severity of these toxicities by evaluating biomarkers indicative of endothelial dysfunction. In a prospective study performed by Moreno-Castaño et al., the levels of several biomarkers between healthy controls and patients undergoing therapy with anti-CD19 or anti-B-cell maturation antigen (BCMA) CAR-T cells complicated by CRS and/or ICANS were compared [46]. Samples were collected: before the CAR-T cell infusion (baseline), 24–48 h after (24 h-INF), during the onset of CRS or ICANS (Tox-onset), and 24–48 h after the administration of immunomodulatory agent (mainly tocilizumab in CRS cases and dexamethasone in ICANS) (post-IMT). Serum markers indicative of endothelial dysfunction, including soluble vascular cell adhesion molecule 1 (sVCAM-1), soluble TNF receptor 1 (sTNFRI), thrombomodulin (TM), soluble C5b-9 (sC5b-9), VWF: Ag and angiopoietin-2 (α2-AP) were significantly higher in CAR-T cell patients at all the time points compared to healthy controls, while ADAMTS-13 activity (A13) was decreased in CAR-T recipients. Notably, these endotheliopathy biomarkers were significantly elevated even at the baseline samples of the treated population [46]. In a phase 1 clinical trial in which children and young adults with relapsed/refractory CD22+ hematologic malignancies treated with anti-CD22 CAR-T cells were included, Jess J. et al. examined the hematologic toxicity profile of CAR-T cells and associations with neurotoxicity [48]. They found that on day 10 (D10) after CAR-T infusion, treated patients who presented with coagulopathy had higher levels of ANG-2 (6411 vs. 3944 pg/mL, *p* = 0.015) and increased ANG-2: ANG-1 ratio (6.25 vs. 3.30, *p* = 0.014) as compared to the rest of the patient cohort. On the other hand, those with coagulopathy displayed lower levels of vascular endothelial growth factor (VEGF) on D10 (10.90 vs. 28.10 pg/mL, *p* = 0.0019) and day 14 (D14) (21.60 vs. 30.15 pg/mL, *p* = 0.011). The definition of coagulopathy was based on the following parameters: presence of bleeding events, increase in D-dimer levels, prothrombin time (PT) values, and nadir of fibrinogen values, which were incorporated in the calculation of a score. Higher levels of LDH (*p* = 0.0005) were observed in patients with coagulopathy, while no statistically significant differences were reported in anticoagulant proteins C and S, or factor VIII, antithrombin, vWF antigen, or vWF activity [48]. Moreover, Ang2 levels on D14 were higher in patients who developed ICANS (5335 vs. 3725 pg/mL, *p* = 0.026) in comparison to those who did not [48].

A correlation between biomarkers indicative of endothelial dysfunction and the severity of CRS was identified after evaluation of a cohort of 133 adult patients with R/R B-ALL, CLL, or NHL who received LD followed by infusion of anti-CD19 CAR-T cells [49]. Interestingly, the data reported in this study, conducted by Hay et al., suggested that preexisting endothelial activation might be a previously unrecognized risk factor for severe CRS. In detail, they showed that patients who developed severe CRS [grade ≥ 4, according to the National Cancer Institute Common Terminology Criteria for Adverse Events (CTCAE v4.0)] exhibited significantly elevated levels of Ang-2, Ang-2:Ang-1 ratio, and VWF at the peak of blood CAR-T expansion, as compared to both subjects without CRS (*p* = 0.02, *p* < 0.0001, *p* = 0.01, respectively) and patients with mild CRS (grade 1–3) (*p* = 0.002, *p* = 0.0007, *p* = 0.002, respectively) [49]. Notably, increased serum VWF, as well as increased Ang-2:Ang-1 ratio, before both LD (*p* = 0.06, *p* = 0.05, respectively) and on day 1 after CAR-T infusion (*p* = 0.01, *p*-0.01, respectively), predicted severe CRS [49]. The predictive role of increased serum VWF levels was maintained on samples collected on the day before CAR-T cell infusion (*p* = 0.03 vs. *p* = 0.29 for Ang-2:Ang1 ratio) [49]. The association between endothelial activation and neurotoxicity development in CAR-T cell-treated patients was assessed by Gust and colleagues, after evaluation of data that concerned 133 adults who received CD19-specific CAR containing a 4-1BB costimulatory domain [38]. In similarity to studies focused on CRS, they showed that the serum Ang-2 concentration (*p* = 0.0003) and the Ang-2:Ang-1 ratio (*p* = 0.0014) were higher in patients with grade ≥ 4 neurotoxicity compared with those with grade ≤ 3 neurotoxicity. Over and above that, to confirm the presence of in vivo endothelial activation in patients with severe neurotoxicity, they proceeded to VWF measurements. As observed, grade ≥4 neurotoxicity was associated with higher serum concentrations of VWF, which in some patients was 4- to 5-fold higher than those observed in serum from patients with grade 0–3 (*p* = 0.004) [38]. On the other hand, high-molecular-weight (HMW) VWF and the ADAMTS13: VWF ratio were found to be decreased during grade ≥4 neurotoxicity (grade ≥ 4 vs. 0–3; ~2% vs. ~9%; *p* = 0.0149 and grade ≥ 4 vs. 0–3; 26.4% vs. 35.2%; *p* = 0.007, respectively).

In sequence, Hong et al. reported clinical and laboratory findings from patients with CD19-R/R ALL after receiving CAR-T cell therapy, aiming to identify modifications in endothelial activation-related molecules that could be characterized as biomarkers associated with CRS [50]. Peripheral blood samples were collected before LD, before CAR-T infusion, and in predetermined intervals following treatment. The levels of VWF, Ang-1, Ang-2, the Ang-2: Ang-1 ratio, and soluble adhesive molecules (sE-selectin, sVCAM-1, and sICAM-1) were compared between treated patients (n = 30) and healthy controls n = 7), as well as between patients with mild (n = 24) and severe CRS (n = 6) [50]. Mild CRS included cases graded 1–3, while severe CRS comprised cases graded 4–5 according to consensus criteria proposed by Lee et al. [51]. Compared to the patients who experienced mild CRS after CAR-T therapy, peak levels of VWF (*p* < 0.01), Ang-2 (*p* < 0.01), Ang-2:Ang-1 ratio (*p* < 0.01), sE-selectin (*p* < 0.01), and sICAM-1 (*p* < 0.01) in the first month were higher in patients with severe CRS, while the nadir level of Ang-1 was lower (*p* < 0.05). Notably, peak levels of these biomarkers corresponded to the peak of CRS, while nadir levels coincided with the resolution of toxicity. It is worth noting that this study also highlights a correlation between the levels of endothelial dysfunction markers and end-organ damage: Elevated levels of creatinine, blood urea nitrogen, aspartate transaminase, and alanine transaminase values—indicating renal and hepatic dysfunction—were found to be significantly associated with increased levels of endothelial activation markers [50].

Moreover, in another prospective cohort study performed by Gust et al., which included 43 patients aged 1 to 25 who received CD19-directed CAR-T cells for ALL, no statistically significant differences in the levels of VEGF-A, ANG-1, ANG-2, or ANG-2: ANG-1 ratio were observed between patients with neurotoxicity (n = 19) and those without (n = 24) [52]. This finding was unanticipated based on results reported in other studies and could be related to a confounding role of CRS, which was present in almost all of the patients involved. Another possible explanation could be the diverse pathophysiology of the pediatric population compared to the adult population [52]. However, more data regarding the role of endothelial injury in pediatric CAR-T cell recipients are essential, given the limited number of studies in the field.

Soluble urokinase-type plasminogen activator receptor (suPAR), a non-specific biomarker of inflammation, has been suggested as a prognostic marker for various acute and chronic clinical entities, such as acute pancreatitis and autoimmune rheumatic disorders [53,54,55]. SuPAR, along with growth/differentiation factor 15 (GDF-15)—a growth factor expressed under conditions of oxidative stress—and the complement activation end-product soluble C5b-9 (sC5b-9), were identified by our group as promising markers of endothelial injury in CAR-T cell recipients [56,57,58]. During the previous year, we published data prospectively collected from 45 patients with B-cell lymphomas and ALL who underwent treatment with axicabtagene ciloleucel, tisagenlecleucel, or brexucabtagene autoleucel [56]. That study evaluated the levels of sC5b-9, SuPAR, and GDF-15 in the treated population before LD administration and compared them with the levels of these markers in 20 healthy controls [56]. As shown, baseline sC5b-9, SuPAR, and GDF-15 levels were significantly elevated in CAR-T cell therapy recipients (median sC5b-9: 209.9 ng/mL, range: 99.9–749.7, *p* < 0.001; median SuPAR: 3.7 ng/mL, range: 2–31.2, *p* < 0.001; median GDF-15: 2807.5, range: 569.2–8655, *p* < 0.001). However, levels of suPAR and GDF-15 measured before LD administration did not differ among the patients who developed severe CRS and/or ICANS post-infusion [56]. A longer follow-up may be required to further investigate the potential role of these substances as predictors of endothelial damage and CAR-T toxicity.

The findings of the various studies are summarized in Table 2. The variability in the results of these studies underscores the need for further investigation to better characterize the behavior of endothelial dysfunction markers in CAR-T cell therapy patients. Moreover, it should be highlighted that in some studies, before the publication of the ASTCT grading scale for CRS and ICANS severity, several other grading criteria have been used, a fact that might explain the difference in the findings between the studies. Rigorous validation in multiple cohorts is essential prior to demonstrating the clinical applicability of biomarkers related to endothelial dysfunction. Such progress is expected not only to refine the evaluation methodology for complication risk development after CAR-T treatment but also to allow early recognition and establishment of efficient prophylactic interventions of CAR-T cell-related toxicities. In the era of personalized medicine, earlier recognition and pre-emptive intervention will make these toxicities less challenging to manage.

### 4.2. Mechanisms of Endothelial Injury in CAR-T Cell Recipients

#### 4.2.1. Complement Activation

Levels of sC5b-9, a marker of terminal complement pathway activation, have been found to be increased in CAR-T cell recipients (calculated before LD administration) as compared to healthy controls. In addition, sC5b-9 levels have been found to be elevated in patients who develop CRS/ICANS in comparison to healthy controls, and have been associated with the toxicity severity [46,56]. The role of the modified ham test, a diagnostic test initially developed for aiding the diagnosis of atypical hemolytic syndrome (a complement-mediated disorder) in the identification of complement activation in the post-infusion period, should be evaluated, comparing not only CAR-T cell recipients with healthy controls but also patients developing CRS, ICANS, or ICAHT of different grading [59]. As has been shown, complement activation is considered a diagnostic hallmark in TA-TMA, an endothelial injury syndrome following HCT [60]. To our knowledge, two TMA cases have been described in the post-infusion period in the CAR-T cell therapy setting [61]. High clinical suspicion and attention are crucial to better identify and understand this possible rare complication after CAR-T cell immunotherapy. As has been described in TA-TMA, cytokine release, especially of interferons, potentially contributes to complement activation [62]. Nevertheless, more data are needed to confirm the dysregulation of the complement system in CAR-T cell recipients.

#### 4.2.2. Endothelial Activation

Markers of endothelial damage, such as VCAM-1, ICAM-1, E-selectin, Ang-2, Ang-1, and Ang-2:Ang-1 ratio, have been found to be increased in patients with severe CRS and/or ICANS compared to those with mild or without toxicities, in several prospective studies [38,46,48,49,50,52]. CRS and the underlying storm of cytokines and other pro-inflammatory molecules result in endothelial dysfunction. In the field of allo-HCT, interestingly, Ang-2 levels, at the time of TA-TMA diagnosis, have been associated with poor outcomes [63]. The binding of Ang-2 on tyrosine kinase receptor Tie2 leads to endothelial cell apoptosis and release of proinflammatory cytokines, while VCAM-1, ICAM-1, and E-selectin induce the migration and adhesion of inflammatory cells [64]. Thus, a vicious cycle between endothelial injury and inflammation is established, which has a crucial role in the development of CRS and ICANS manifestations. The role of these markers in the prediction of CAR-T cell recipients’ long-term outcomes, such as cardiovascular disease, should be further evaluated. For this aim, longitudinal follow-up is essential.

#### 4.2.3. Procoagulant State

**Figure 1:**
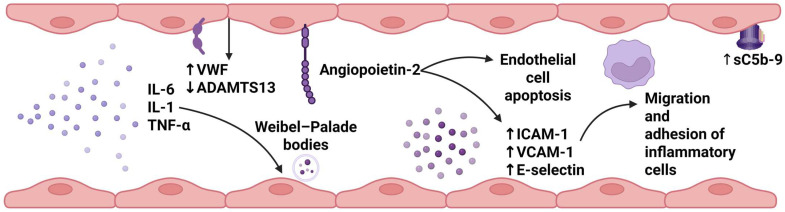
Endothelial injury in CAR-T cell recipients. Various markers of endothelial injury have been investigated in CAR-T cell recipients, including markers of complement activation, such as soluble C5b-9, endothelial dysfunction (Ang-2, VCAM1, ICAM-1, E-selectin), inflammation, and thrombosis (VWF, ADAMTS13). The expression of these endothelial injury markers has been identified as impaired in CAR-T cell recipients, not only when compared with healthy controls but also when compared between patients with severe CRS/ICANS and those with mild toxicities or without toxicities. ↑: increased; ↓: decreased; ADAMTS13 = a disintegrin and metalloproteinase with a thrombospondin type 1 motif member 13; CAR-T = chimeric antigen receptor-T; ICAM-1 = intercellular adhesion molecule; IL-6 = interleukin-6; IL-1 = interleukin-1; sC5b-9 = soluble C5b-9. TNF-α = tumor necrosis factor-α; VCAM-1 = vascular adhesion molecule; VWF: von Willebrand factor (created in BioRender. Evangelidis, P. (2025) https://BioRender.com/bwzsi9g, accessed on 11 May 2025).

Increased serum levels of thrombomodulin and VWF antigen, as well as decreased activity of ADAMTS13 (compared to healthy controls) have also been reported in the post-CAR-T cell infusion period [38,46]. VWF is a plasma glycoprotein that has a pivotal role in hemostasis, promoting platelet adhesion, clot formation, and stabilization of factor VIII [65]. At the same time, ADAMTS13, a metalloprotease, is responsible for the cleavage of ultra-large VWF into smaller molecules [66]. Decreased ADAMTS13 activity has been recognized in several clinical entities: an activity level of less than 10% is essential for the confirmation of TTP diagnosis in patients with evidence of hemolysis and thrombocytopenia [67,68]. Future studies should further evaluate the role of ADAMTS13 and VWF axis dysregulation during the post-infusion period after treatment with CAR-T cells. Moreover, multicenter collaboration and real-world data can be helpful for a better understanding of the associations between thrombotic/bleeding events in these patients. The markers of endothelial injury in CAR-T cell immunotherapy recipients are summarized in Table 3 and illustrated in Figure 1.

## 5. Endothelial Injury Indices in the Prediction of CAR-T Cell Immunotherapy Outcomes

The Endothelial Activation and Stress Index (EASIX), which is calculated by common laboratory values (EASIX; LDH [units per liter] × creatinine [milligrams per deciliter]/platelets [PLTs] [×10^9^ per liter]), has been suggested as a marker of endothelial injury in allo-HCT recipients, while several studies have validated the predictive role of this score in overall survival (OS) and other outcomes in this patient population [71,72,73]. Given the common pathophysiological mechanisms between endothelial injury syndromes post-allo-HCT, such as TA-TMA, and toxicities following CAR-T cell immunotherapy, the EASIX score has been examined as a marker not only of endothelial injury but also of OS and outcome in CAR-T cell recipients [74]. Pennisi and colleagues were the first to examine the predictive role of EASIX scores in the prediction of CAR-T cell-related toxicities [75]. In this study, EASIX, modified EASIX (mEASIX; LDH [units per liter] X C-reactive protein [milligrams per deciliter]/PLTs [×10^9^ per liter]), and simplified EASIX (sEASIX; LDH [units per liter]/PLTs [×10^9^ per liter]) were calculated before CAR-T cell infusion (start of LD and day −1), and early post-CAR-T cell infusion (day +1 and +3), including the day of onset of CRS. They confirmed the role of EASIX/sEASIX/mEASIX scores as predictors for severe CRS (grade ≥ 3) calculated prior to LD, at day −1, day +1, and day +3. On the other hand, only mEASIX values at day +3 showed discriminatory ability for severe ICANS, grade ≥ 3. Further analysis demonstrated that patients with higher mEASIX scores at the aforementioned timepoints were less likely to achieve complete response by day 90 after infusion.

Similarly, Korell et al. investigated the predictive role of endothelial injury indices in 214 CAR-T cell recipients (93 in the training cohort and 121 in the validation cohort) [69]. It was reported that EASIX calculated prior to the LD (pLD) predicted the development of grade ≥ 3 CRS and/or ICANS in both training (*p* = 0.001) and validation cohorts, while a pLD EASIX cutoff value > 4.67 in the training cohort was associated with a 4.3-fold increased odds ratio for severe complications post-infusion (*p* = 0.006). Additionally, markers of endothelial injury, including suppression of tumorigenicity 2 (ST2), Ang-2, soluble thrombomodulin, and IL-8, measured on day 7 post-CAR-T cell infusion, were correlated with pLD EASIX. In the study of Greenbaum et al., pLD EASIX score combined with ferritin values (EASIX-F) was associated with the development of CRS grade 2 to 4 in 171 of axicabtagene ciloleucel recipients [76]. Specifically, the modified EASIX-F was able to identify three risk groups with a cumulative incidence of CRS grades ≥ 2 of 74% (HR, 3.6; *p* < 0.001), 51% (HR, 2.1; *p* = 0.025), and 29% (reference), supporting the role of the implicated laboratory parameters in CAR-T-related toxicities. In addition, patients classified to the highest EASIX-FC risk group showed a trend for increased incidence of ICANS grade 3 to 4 that reached 60% (HR, 3.8; *p* < 0.001).

To further explore the role of EASIX and mEASIX in a real-world setting, our group recently published data from a retrospective multicenter study concerning 90 patients who received commercially available CAR-T cell therapy for R/R B-ALL or lymphoma [77]. In our study, EASIX and mEASIX scores were calculated at different time points (pLD, day of infusion, day 14 post-infusion) [77]. Based on our analysis, pLD mEASIX was able to predict the development of CRS grade ≥ 3 in ROC analysis, contrary to severe ICANS, which was not associated with EASIX markers. Moreover, we were among the first to show that EASIX and mEASIX scores calculated at day 14 following CAR-T infusion were associated with OS after a median follow-up of 6.6 months (range, 1–42 months). At the same time, EASIX14 and m-EASIX14 predicted the risk of death in the ROC curves. In another study published by our group, the association between markers of endothelial injury, such as suPAR, GDF-15, and sC5b-9, with EASIX, mEASIX, and sEASIX scores was evaluated. Multivariate analysis derived from data concerning 45 patients treated with CAR-T cell therapy demonstrated that baseline suPAR levels were correlated with mEASIX at the same time point (*p* < 0.001). In addition, baseline suPAR and GDF-15 levels were associated with EASIX score at day 14 (*p* = 0.017 and *p* < 0.001, respectively) [56]. Notably, sEASIX scores calculated on the day of CAR-T cell infusion and day 14 after infusion were highlighted as predictors of OS in this patient cohort (*p* = 0.039 and *p* = 0.016, respectively).

In another retrospective study that included 84 individuals treated with axi-cel, Acosta-Medina and colleagues failed to demonstrate a significant association of EASIX with CRS [78]. In this study, a complete laboratory evaluation enabled calculation of both EASIX and mEASIX scores prior to LD and on the day of CAR-T cell infusion. Following further analysis, it was found that patients with higher pLD-EASIX (*p* = 0.04) and infusion-EASIX (*p* = 0.008) were more likely to develop severe ICANS of grade ≥ 3. There was no association between EASIX scores and CRS development. In terms of treatment, increasing pLD-EASIX (*p* = 0.019) and infusion-EASIX (*p* = 0.001) were both correlated with higher cumulative doses of steroids for management of CRS/ICANS. Concerning mEASIX, infusion-mEASIX was significantly associated with increased risk for severe ICANS (*p* = 0.034) and with increased cumulative steroid dosing (*p* = 0.010). Notably, only the mEASIX was able to categorically predict high- and low-risk patients (cutoff ≥4), with patients classified to the high-risk group being four times more likely to develop grade 3–4 ICANS (*p* = 0.034). They reported that the lack of correlation between EASIX and CRS in this patient cohort was attributed to the limited rates of observed severe CRS (n = 2), the exclusive use of CD28 costimulatory-based CAR-T products, and the inclusion of a limited population with transformed indolent B-cell lymphomas, in addition to the relatively small sample size of the cohort [78].

De Boer and colleagues studied 154 lymphoma patients treated with axi-cel and reported that EASIX derivatives calculated prior to LD were significantly higher (EASIX, *p* < 0.01; mEASIX, *p* = 0.03; sEASIX, *p* = 0.02) for patients with grade ≥ 2 ICANS, but no associations were identified for CRS grade ≥ 2 and/or ICANS grade ≥ 3 [79]. Nevertheless, the predictive performance of these scores was only moderate [79].

As mentioned above, the pro-inflammatory cytokine IL6 is a contributor to vascular endothelium instability. Another emerging biomarker that seems to be related to CAR-T system activation is phosphorus, and studies revealed that hypophosphatemia often precedes the onset of CRS/ICANS [80,81]. Recently, Barker et al. described phosphorus mEASIX (*p*-mEASIX) and IL-6 mEASIX (IL-6-mEASIX), in which phosphorus and IL-6 values, respectively, are incorporated [82]. It was reported that P-mEASIX calculated on day +1 post-infusion was characterized by improved predictive capabilities of ICANS compared to mEASIX score at the same time point. Furthermore, both P-mEASIX and IL-6-mEASIX predicted CRS grade ≥ 2 development. However, IL6-m-EASIX showed the largest increase in AUC for ROC analysis compared to the other scores on day +3, which reflects a greater enhancement in the discriminatory function for predicting CRS progression to grade ≥ 2.

The role of EASIX score and derivatives as predictors of toxicities following CAR-T cell treatment has also been evaluated in the pediatric population. In the retrospective study of Zandaki et al., median EASIX scores at several time points were higher for patients who exhibited severe CRS (−5, 0, and +3, *p* < 0.05 in all comparisons), although the dispersion of EASIX score decreased over time [83]. Regarding mEASIX, scores were consistently higher for patients who experienced severe CRS (days −5, 0, and +3, *p* < 0.05 in all comparisons) than those with mild or no CRS. For individuals who developed ICANS, EASIX scores were consistently higher regardless of the ICANS grading as compared to patients who never developed ICANS of any grade compared to the rest of the study population. Interestingly, median mEASIX scores showed a noticeable rise only for patients with severe ICANS [83]. Both scores demonstrated stronger predictive performance for severe CRS than any CRS. mEASIX scores had consistently higher AUCs at each evaluable time, whereas the best performance was demonstrated on day +3.

Data regarding the prognostic role of EASIX scores in CRS/ICANS development in multiple myeloma (MM) CAR-T cell recipients are conflicting. Tomasik et al. included in their retrospective study 69 patients with MM and light-chain amyloidosis (AL) who were treated with anti-BCMA (NXC-201) CAR-T cell products [84]. As demonstrated, in this specific cohort, EASIX and sEASIX scores, as calculated at different time points prior to LD and infusion, failed to predict the development of CRS grade ≥ 3. Of course, larger cohorts may be required in order to reach safer conclusions regarding the potential of using the EASIX score and derivatives in the clinical setting. Moreover, future studies should examine the role of these scores in the prediction of outcomes in plasma cell disorder patients who undergo CAR-T cell immunotherapy with the commercially available anti-BCMA constructs.

CAR-T therapy has been proven to serve as a curative option for several patients with complicated R/R/B-cell malignancies; however, application of this strategy to acute myeloid leukemia (AML) patients is restricted due to the heterogeneity of AML cells and the lack of a specific target that would minimize the off-target effects [85]. The human C-type lectin-like molecule 1 (CLL1) might be a promising target, as it is primarily expressed in leukemia stem cells and blasts but is absent in healthy tissues and hematopoietic stem cells [86]. In a single-arm clinical trial, Zhao et al. enrolled 33 patients with R/R AML who received immunotherapy with anti-CLL1 CAR-T cells [87]. EASIX, mEASIX, and sEASIX scores were calculated at the following time points: the day before the administration of LD (day BL), the day before the infusion of CAR-T cell products (day −1), the day after CAR-T cell infusion (day +1), and 2 days afterwards (day +3). The researcher reported statistically significant differences in these scores between patients who developed grade 3/4 (severe) CRS/ICANS and the rest of the study participants. Specifically, severe CRS/ICANS was correlated with EASIX scores on day BL, −1, +1, and +3 sEASIX scores on day BL, −1, +1, and +3, and mEASIX score on day −1. Moreover, in ROC analysis, the research reports significant correlations between these scores and toxicity occurrence. Additionally, the authors examined the possible associations between the above-described scores and the peak levels of biochemical and inflammatory markers. Interestingly, IL-6 peak levels were associated with EASIX on day −1, +1, and +3; sEASIX on day BL, −1), +1, and +3; and mEASIX on day −1 [87]. More data regarding the prognostic role of EASIX scores in patients who have received CAR-T cell therapy for R/R AML are essential, while multicenter collaboration is crucial for this purpose.

Beyond the prediction of post-infusion toxicities, EASIX indices have been used as prognostic scores for other CAR-T-associated complications, such as hematological toxicities and bleeding events. Wang and colleagues showed that pLD EASIX and IL-10 levels were identified as significant risk factors for bleeding events post-infusion [88]. In another study, pLD mEASIX score was correlated with increased aPTT values, fibrinogen, D-dimer, factor VIII (FVIII), and VWF levels, and decreased antithrombin levels, while an mEASIX value greater than 6.89 predicted DIC during the two weeks following infusion (ROC analysis, sensitivity 53%, specificity 88%) [70]. Interestingly, pLD EASIX score has been identified as a risk factor for severe ICAHT, aplastic neutrophil recovery pattern, and severe late-onset infections post-infusion in CAR-T cell recipients with R/R MM [89]. In Table 4, we provide an overview of the associations between predictive endpoints and EASIX derivatives.

## 6. Therapeutic Implications

### 6.1. Statins

Statins have long been proposed as endothelial protective agents based on their immunomodulatory and anti-inflammatory properties [90]. Moreover, statins can decrease the levels of oxidized low-density lipoprotein and the activity of nicotinamide adenine dinucleotide phosphate oxidase, resulting in a reduction in reactive oxygen species (ROS) [91]. Thus, via indirect signaling, the coupling of endothelial nitric oxide is enhanced. Based on these data, statins may be used as a potential therapeutic agent in the field of CAR-T cell therapy-related toxicities. Adroja and colleagues described in an abstract presented at the American Society of Hematology (ASH) 2024 annual meeting the impact of statins on outcomes of patients with B-cell lymphomas treated with CD19 CAR-T cell products [92]. Interestingly, statin use was correlated with significantly improved OS and progression-free survival. Nevertheless, the incidence of CRS and ICANS was not different between statin and no-statin-exposed patients. Yu et al. analyzed the data from 3001 patients who received CAR-T cell therapy for hematological malignancies and compared the outcomes between those who had statin exposure (n = 1040) and those who did not (n = 1961, control group) [93]. It was reported that statin use was associated with an 8.6% improvement in 5-year OS compared to the control group (*p* < 0.001). However, no significant differences were found in rates of CRS, ICANS, or infections between the two groups. Preliminary data from an ongoing clinical trial (NCT04514029), which examined the safety and efficacy of simvastatin (5 days before apheresis to day +30 post-infusion) and intrathecal dexamethasone (days −1 and +6 in relation to CAR-T cell infusion) in CAR-T cell patients, showed that the incidence of ICANS was 26.7%, with grade ≥3 ICANS in only 13.3%, lower than historical cohort rates, especially among those who received both interventions [94]. More data regarding the role of statins in this patient population is crucial.

### 6.2. Defibrotide

Defibrotide has been recognized as an endothelial protective agent with several anti-inflammatory, antithrombotic, antioxidant, and antiadhesion properties, enhancing vascular stability and angiogenesis [95]. Additionally, defibrotide has been found to reduce endothelial activation by affecting the release of inflammatory mediators and ROS production and the activity of nitric oxide synthase. Defibrotide administration is standard of care for the management of patients with hepatic sinusoidal obstruction syndrome/vein occlusive disease (SOS/VOD), an endothelial injury syndrome observed in allo-HCT recipients [96]. In a phase 2 trial by Jacobson et al., defibrotide was evaluated as a prophylactic agent for ICANS patients with R/R large B-cell lymphoma treated with axicabtagene ciloleucel. While defibrotide showed a reduction in neurotoxicity rates (∼50%) compared to historical cohort data, it was not statistically significant, leading to early termination of the study [97].

### 6.3. TNF-α and IL-1β Blockade

Another important contributor to endothelial activation and injury post-CAR-T cell infusion includes TNF-α, released from CAR-T cells, and IL-1β from myeloid cells, contributing to the development of CRS and ICANS, as described above [98]. Blocking these pathways with adalimumab, an anti-TNF-α monoclonal antibody, and with anti-IL-1β antibodies has been found to significantly reduce endothelial activation in experimental models [98]. These findings highlight the therapeutic potential of targeting TNF-α and IL-1β signaling to mitigate endotheliopathies following infusion. Nevertheless, more research in the field is essential.

## 7. Conclusions—Future Perspectives

Toxicities following CAR-T cell infusion, such as CRS and ICANS, result from the complex interplay between macrophages, T cells, and innate immunity, leading to endothelial dysfunction and injury. Various markers indicative of endothelial injury have been studied in these patients. Significant findings have identified the EASIX score and derivatives as predictors of outcome, OS, and progression-free survival in CAR-T-treated individuals. Specifically, the EASIX score calculated before the administration of LD and mEASIX estimated on the day of infusion have emerged as potential markers for the prediction of severe CRS and ICANS in this patient population. Thus, they could be used as potentially useful markers for the identification of patients who are at great risk for the development of severe CRS and ICANS necessitating close monitoring and, possibly, early intervention. Interestingly, the predictive role of EASIX indices has been shown in patients who have received various CAR-T cell products, both commercially available ones and those under investigation in clinical trials. Given the role of endothelial dysfunction in cardiovascular disease (CVD), it is crucial to monitor CAR-T cell therapy survivors closely for CVD development.

Future research perspectives include:

- Conducting real-world studies to explore the role of endothelial injury indices in predicting infectious events, malignancy relapse, and severe cytopenias post-infusion.
- Performing meta-analyses, particularly individual patient data meta-analyses, to elucidate the role of these scores.
- Investigating ADAMTS13 activity, von Willebrand factor (VWF), and complement system activation in patients with severe toxicities through translational research approaches.
- Undertaking multicenter collaborative studies to identify cases of transplant-associated thrombotic microangiopathy (TMA) post-infusion.
- Exploring the associations between EASIX scores, endothelial injury markers, and cognitive outcomes.
- Studying pre-infusion genetic susceptibility to endothelial injury syndromes.
- Assessing microcirculation with non-interventional methods in long-term CAR-T cell therapy survivors.
- Investigating endothelial injury post-infusion in patients receiving CAR-T cell therapy for autoimmune diseases and examining the role of EASIX scores in these patients.

## Figures and Tables

**Table 1:** CRS and ICANS grading based on ASTCT consensus as proposed by Lee et al. [26].

Grade	CRS	ICANS
I	Fever ≥ 38.0 °C	ICE score: 7–9 Depressed level of consciousness, awakens spontaneously
II	Fever ≥ 38.0 °C with hypotension requiring IV fluids but not requiring vasopressors and/or hypoxia requiring oxygen via LFNC (≤6/L minute) ^1^	ICE score: 3–6 Depressed level of consciousness, awakens to voice
III	Fever ≥ 38.0 °C with hypotension requiring one vasopressor (±vasopressin) and/or hypoxia requiring oxygen via HFNC (≥6/L minute), facemask, non-breather or venturi mask ^1^	ICE score: 0–2 Depressed level of consciousness, awakens to tactile stimulus Seizure: Any clinical seizure, focal or generalized, that resolves rapidly, or nonconvulsive seizures on EEG that resolve with intervention Cerebral edema: Focal/local edema on neuroimaging
IV	Fever ≥ 38.0 °C with hypotension requiring multiple vasopressors (not vasopressin) and/or hypoxia requiring positive pressure oxygenation (e.g., CPAP, BiPAP, intubation and mechanical ventilation ^1^	ICE score: 0 Depressed level of consciousness: Unarousable patient, or requiring vigorous or repetitive tactile stimuli to arouse or stupor or coma Motor findings: Deep focal motor weakness such as hemiparesis or paraparesis Elevated ICP: Diffuse cerebral edema on neuroimaging, decerebrate or decorticate posturing, cranial nerve VI palsy, papilledema, or Cushing’s triad

^1^ Hypoxia should not be attributed to any other causes. ASTCT = American Society of Transplantation and Cellular Therapy; CRS = cytokine release syndrome; HFNC = high-flow nasal cannula; ICANS = immune effector cell-associated neurotoxicity syndrome; ICE = immune effector cell-associated encephalopathy; ICP = increased intracranial pressure; LFNC = low-flow nasal cannula.

**Table 2:** Overview of the studies investigating endothelial injury markers in CAR-T cell recipients.

First Author, Year, Reference	Study Design	Control Group	Patient Population	Markers of Endothelial Injury	Significant Findings
Moreno-Castaño et al., 2023 [46]	Prospective study	Healthy controls (n = 49)	CAR-T cell recipients who developed CRS/ICANS (n = 19)	s-VCAM1, STNFRI, TM, ST2, ADAMTS13 activity, Ang-2, NETs, sC5b-9, VWF antigen, a2-AP, PAI-1 Ag	- All the markers were significantly increased in patients with CRS/ICANS compared to healthy controls - DAMTS13 activity was significantly higher in the control group
Jess et al., 2023 [48]	Prospective study	Patients without ICANS (n = 33) or coagulopathy (n = 30) post-infusion	Patients who developed ICANS (n = 20) or coagulopathy (n = 18) post-infusion	Ang-2, Ang-2:Ang-1 ratio, fibrinogen nadirs, protein C, S, factor VIII, antithrombin, VWF antigen and activity, VEGF	- In patients with ICANS, Ang-2 and Ang-2:Ang-1 ratio were significantly increased (day 10 and day 14 post-infusion) - There were no significant differences in protein C, S, VIII, antithrombin, or VWF antigen activity and Ang1 levels between patients with and without coagulopathy
Hong et al., 2021 [50]	Prospective study	Healthy controls (n = 7) and patient with mild CRS (n = 24)	CAR-T cell recipients (n = 30) and patients with severe CRS (n = 6)	VWF, Ang-1, Ang-2, Ang-2:Ang-1 ratio, sVCAM, sICAM-1, E-selectin ^1^	Peak levels of these markers were significantly elevated in post-CAR-T cell therapy patients and in patients with severe CRS, compared to healthy controls and those without severe CRS, respectively
Hay et al., 2017 [49]	Phase 1 clinical trial	CAR-T cell recipients with CRS grade 1–3 (n = 51)	Patients experiencing severe CRS, defined as grade ≥ 4 (n = 9)	VFW, Ang-2, Ang-1 and Ang-2:Ang-1 ratio	The levels of the assessed markers were significantly associated with CRS severity
Gust et al., 2019 [52]	Prospective study	CAR-T cell recipients without ICANS (n = 24)	CAR-T cell recipients with ICANS (n = 19)	VEGF-A, Ang-1, Ang-2	No statistically significant differences were identified between the two groups
Gust et al., 2017 [38]	Prospective study	CAR-T cell recipients with grade 0–3 ICANS (multiple subgroups were assessed for comparison)	Patients developing grade ≥ 4 ICANS	Ang-1, Ang-2, Ang-2:Ang-1 ratio, VWF (including LMW and HMW VWF), ADAMTS13:VWF ratio	- Ang-1, Ang-2, Ang2:1 ratio, and VWF were significantly increased in patients with grade ≥ 4 ICANS - Ang-2:Ang-1 ratio pLD was significantly elevated in patients with severe neurotoxicity post-infusion - Increased LMW VWF, decreased HMW VWF, and decreased ADAMTS13: VWF ratio were observed in patients with grade ≥ 4 ICANS
Gavriilaki et al., 2024 [56]	Prospective study	Healthy controls (n = 20)	CAR-T cell recipients (n = 45)	suPAR, GDF-15, sC5b-9 ^2^	These biomarkers were significantly higher in patients treated with CAR-T cells

^1^ The levels of these markers were compared between patients who received CAR-T cell therapy (n = 30) and healthy controls (n = 7), as well as between patients with mild (n = 24) and severe CRS (n = 6). ^2^ These markers were assessed pLD. a2-AP = α2-antiplasmin activity; ADAMTS13 = a disintegrin and metalloproteinase with a thrombospondin type 1 motif member 13; Ag = antigen; Ang-1 = angiopoietin-1; Ang-2 = angiopoietin-2; CAR-T = chimeric antigen receptor-T; CRS = cytokine release syndrome; GDF-15 = growth/differentiation factor 15; HMW = high molecular weight; ICANS = immune effector cell-associated neurotoxicity syndrome; LMW = low molecular weight; NETs = neutrophil extracellular traps; PAI-1 = plasminogen activator inhibitor-1; pLD = prior the administration of lymphopledating chemotherapy; sC5b-9 = soluble C5b-9; sICAM-1 = soluble intercellular adhesion molecule-1; ST2 = soluble suppression of tumorigenesis-2; STNFRI = soluble tumor necrosis factor receptor I; suPAR = soluble urokinase-type plasminogen activator receptor; s-VCAM1 = soluble vascular cell adhesion molecule 1; TM = thrombomodulin; VEGF = vascular endothelial growth factor; VWF = von Willebrand factor.

**Table 3:** Markers of endothelial injury in CAR-T cell immunotherapy recipients.

Category	Marker of Endothelial Injury	Pathophysiological Role	Significant Findings	References
Complement	sC5b-9	- Innate immunity response - Cell damage and lysis	Increased in CAR-T cell recipients compared to normal controls and in CRS, ICANS	[46,56]
Endothelium	VCAM-1	- Adhesion of leukocytes to the endothelium - Amplification of the inflammatory cascade	Increased in CRS, ICANS	[46,50]
	ICAM-1	- Adhesion of leukocytes to the endothelium - Amplification of the inflammatory cascade	Increased in CRS and severe CRS	[50]
	E-selectin	- Leukocyte recruitment to inflamed tissues - Endothelial activation	Increased in CRS and severe CRS	[50]
	Ang-2	- Mediator of endothelial activation and destabilization	Increased in CRS and severe CRS	[38,46,48,49,50,69]
Procoagulant molecules	TM	- Endothelial damage	Increased in CRS, ICANS	[46,69]
	ADAMTS13 activity	- Microvascular thrombosis - Organ dysfunction	Decreased in CRS, ICANS	[46]
	VWF	- Endothelial activation - Microvascular thrombosis - Capillary leak and organ dysfunction	Increased in CRS, ICANS Association with severe ICANS	[38,46,70]
Proinflammatory molecules	STNFRI	- Systemic inflammation - Tight junction breakdown	Increased in CRS, ICANS	[46]
	ST2	Disruption of vascular integrity	Increased in CRS, ICANS	[46,69]
	NETs	Systemic inflammation and cytokine surge	Increased in CRS, ICANS	[46]

ADAMTS13 = a disintegrin and metalloproteinase with a thrombospondin type 1 motif member 13; Ang-1 = angiopoietin-1; Ang-2= angiopoietin-2; CAR-T = chimeric antigen receptor-t; EASIX = Endothelial Activation and Stress Index; ICAM-1 = intercellular adhesion molecule-1; ICANS = immune effector cell-associated neurotoxicity; NETs = neutrophil extracellular traps; sC5b-9 = soluble C5b-9; CRS: cytokine release syndrome; sEASIX = simplified Endothelial Activation and Stress Index; ST2 = soluble suppression of tumorigenesis-2; STNFRI = soluble tumor necrosis factor receptor I; TM = thrombomodulin; VCAM-1 = vascular cell adhesion molecule 1; VWF = von Willebrand factor.

**Table 4:** Overview of the associations between predictive endpoints and EASIX derivatives in CAR-T cell recipients.

Predictive Endpoint	Endothelial Injury Indices	Time Point of Assessment	References
Severe CRS (grade ≥ 3)	EASIX	pLD, infusion, days 1 to 3 post-infusion	[69,75,76,77,82,83]
	mEASIX	pLD, infusion, days 1 to 3 post-infusion	
	EASIX-F	pLD	
	P-mEASIX	Day 1 post-infusion	
	IL6-mEASIX	Day 1 post-infusion	
Severe (grade ≥ 3)	EASIX	pLD, infusion	[69,75,76,78,79,82,83]
	mEASIX	pLD, infusion, day 3 post-infusion	
	EASIX-F	pLD	
	sEASIX	pLD	
	P-mEASIX	Day 1 post-infusion	
Hematological toxicity, DIC, bleeding events	EASIX	pLD	[70,88,89]
	mEASIX	pLD	
Complete response	mEASIX	Days 1 to 3 post-infusion, on CRS onset	[75]
Overall survival	EASIX	Day 14 post-infusion	[56,77]
	mEASIX	Day 14 post-infusion	
	sEASIX	Infusion, day 14 post-infusion	

CAR-T = chimeric antigen receptor-T; CRS = cytokine release syndrome; DIC = disseminated intravascular coagulation; EASIX = endothelial activation and stress index; EASIX-F = endothelial activation and stress index with ferritin; ICANS = immune effector cell-associated neurotoxicity syndrome; IL6-mEASIX = interleukin-6 modified Endothelial Activation and Stress Index; mEASIX = modified EASIX = modified Endothelial Activation and Stress Index; pLD = prior lymphodepleting chemotherapy; P-mEASIX = phosphorus-modified Endothelial Activation and Stress Index; sEASIX = simplified Endothelial Activation and Stress Index.

## Abbreviations

The following abbreviations are used in this manuscript:

AL	Light-chain amyloidosis
AML	Acute myeloid leukemia
ANG-1	Angiopoietin-1
ANG-2	Angiopoietin-2
aPTT	Activated partial thromboplastin
ASTCT	American Society for Transplantation and Cellular Therapy
AUC	Area under the curve
B-ALL	B-cell acute lymphoblastic leukemia
BCMA	B-cell maturation antigen
BTK	Bruton’s tyrosine kinase
CAR-T	Chimeric antigen receptor-T
CLL1	C-type lectin-like molecule 1
CRP	C-reactive protein
CRS	Cytokine release syndrome
DAMPs	Danger associated molecular patterns
EASIX	Endothelial Activation and Stress Index
EASIX-F	Endothelial Activation and Stress Index score combined with ferritin values
ECs	Endothelial cells
EEG	Electroencephalogram
FDA	Food and Drug Administration
FiO2	Fraction of inspired oxygen
FRIDA	Frontal rhythmic intermittent delta activity
FVIII	Factor VIII
GDF-15	Growth/differentiation factor 15
GM-CSF	Granulocyte-macrophage colony-stimulating factor
HCT	Hematopoietic cell transplantation
HLH	Hemolytic lymphohistiocytosis
HMW	High molecular weight
ICAHT	Immune effector cell-associated hematotoxicity
ICAM-1	Intercellular adhesion molecule 1
ICANS	Immune effector cell-associated syndromes
ICE	Immune effector cell-associated encephalopathy
IL-1	Interleukin 1
IL-6	Interleukin 6
IL-6-mEASIX	IL-6 modified Endothelial Activation and Stress Index
INF-γ	Interferon-γ
JAK/STAT	Janus kinase/signal transducers and activators of transcription
LD	Lymphodepleting chemotherapy
LDH	Lactate dehydrogenase
LMW	Low molecular weight
MAPK/NFκB	Mitogen-activated protein kinase/nuclear factor kappa-light-chain enhancer of activated B cells
MAS	Macrophage activation syndrome
mEASIX	Modified Endothelial Activation and Stress Index
MM	Multiple myeloma
MRI	Magnetic resonance imaging
NETs	Neutrophil extracellular traps
NHL	Non-Hodgkin lymphoma
OS	Overall survival
PLTs	Platelets
P-mEASIX	Phosphorus-modified Endothelial Activation and Stress Index
PT	Prothrombin time
R/R	Relapsed/refractory
RES	Reticuloendothelial system
ROC	Receiver operating characteristics
sC5b-9	Soluble C5b-9
sEASIX	Simplified Endothelial Activation and Stress Index
sICAM-1	Soluble intracellular cell adhesion molecule-1
suPAR	Soluble urokinase-type plasminogen activator receptor
sVCAM	Soluble vascular cell adhesion molecule
TA-TMA	Transplant-associated thrombotic microangiopathy
TIE-2	Angiopoietin-1 receptor
TLS	Tumor lysis syndrome
TNF-a	Tumor necrosis factor-a
VCAM-1	Vascular cell adhesion molecule 1
VEGF	Vascular endothelial growth factor
VWF	Von Willebrand factor
